# Specifically neuropathic Gaucher's mutations accelerate cognitive decline in Parkinson's

**DOI:** 10.1002/ana.24781

**Published:** 2016-11-18

**Authors:** Ganqiang Liu, Brendon Boot, Joseph J. Locascio, Iris E. Jansen, Sophie Winder‐Rhodes, Shirley Eberly, Alexis Elbaz, Alexis Brice, Bernard Ravina, Jacobus J. van Hilten, Florence Cormier‐Dequaire, Jean‐Christophe Corvol, Roger A. Barker, Peter Heutink, Johan Marinus, Caroline H. Williams‐Gray, Clemens R. Scherzer, for the International Genetics of Parkinson Disease Progression (IGPP) Consortium, C. Scherzer, B.T. Hyman, A.J. Ivinson, A. Trisini‐Lipsanopoulos, D. Franco, K. Burke, L.R. Sudarsky, M.T. Hayes, C.C. Umeh, J.H. Growdon, M.A. Schwarzschild, A.Y. Hung, A.W. Flaherty, A.‐M. Wills, N.I. Mejia, S.N. Gomperts, V. Khurana, D.J. Selkoe, T. Yi, K. Page, Z. Liao, R. Barker, T. Foltynie, C.H. Williams‐Gray, S. Mason, S. Winder‐Rhodes, R. Barker, C.H. Williams‐Gray, D. Breen, G. Cummins, J. Evans, S. Winder‐Rhodes, J.‐C. Corvol, A. Brice, A. Elbaz, A. Mallet, M. Vidailhet, A.‐M. Bonnet, C. Bonnet, D. Grabli, A. Hartmann, S. Klebe, L. Lacomblez, G. Mangone, F. Bourdain, J.‐P. Brandel, P. Derkinderen, F. Durif, V. Mesnage, F. Pico, O. Rascol, S. Forlani, S. Lesage, K. Tahiri, J.J. van Hilten, J. Marinus, Z. Liao, K. Page, D. Franco, K. Duong, T. Yi, A. Trisini‐Lipsanopoulos, X. Dong, L.R. Sudarsky, S.J. Hutten, S.S. Amr, I. Shoulson, C.M. Tanner, A.E. Lang, M.A. Nalls

**Affiliations:** ^1^ Neurogenomics Lab and Parkinson Personalized Medicine Program, Harvard Medical School and Brigham & Women's HospitalCambridgeMA; ^2^ Ann Romney Center for Neurologic Diseases, Brigham and Women's HospitalBostonMA; ^3^ Biomarkers Program, Harvard NeuroDiscovery CenterBostonMA; ^4^ Department of NeurologyBrigham and Women's HospitalBostonMA; ^5^ Department of NeurologyMassachusetts General HospitalBostonMA; ^6^ Department of Medical GenomicsVU University Medical Center, Neuroscience Campus AmsterdamAmsterdamHZThe Netherlands; ^7^ German Center for Neurodegenerative diseases (DZNE)TübingenGermany; ^8^ John Van Geest Centre for Brain Repair, Department of Clinical NeurosciencesUniversity of CambridgeCambridgeUnited Kingdom; ^9^ Department of Biostatistics and Computational BiologyUniversity of Rochester Medical CenterRochesterNY; ^10^ INSERM, Centre for Research in Epidemiology and Population Health, U1018, Epidemiology of ageing and age related diseasesVillejuifFrance; ^11^ University Paris‐Sud, UMRS 1018VillejuifFrance; ^12^ Sorbonne Université, Université Pierre et Marie Curie Paris 06 UMR S 1127, Institut National de Santé et en Recherche Médicale U 1127 and Centre d'Investigation Clinique 1422, Centre National de Recherche Scientifique U 7225, Institut du Cerveau et de la Moelle Epinière, Assistance Publique Hôpitaux de Paris, Département de Neurologie et de Génétique, Hôpital Pitié‐SalpêtrièreParisFrance; ^13^ Voyager Therapeutics, Clinical DevelopmentCambridgeMA; ^14^ Department of NeurologyLeiden University Medical CenterLeidenThe Netherlands

## Abstract

**Objective:**

We hypothesized that specific mutations in the β‐glucocerebrosidase gene (*GBA*) causing neuropathic Gaucher's disease (GD) in homozygotes lead to aggressive cognitive decline in heterozygous Parkinson's disease (PD) patients, whereas non‐neuropathic GD mutations confer intermediate progression rates.

**Methods:**

A total of 2,304 patients with PD and 20,868 longitudinal visits for up to 12.8 years (median, 4.1) from seven cohorts were analyzed. Differential effects of four types of genetic variation in *GBA* on longitudinal cognitive decline were evaluated using mixed random and fixed effects and Cox proportional hazards models.

**Results:**

Overall, 10.3% of patients with PD and *GBA* sequencing carried a mutation. Carriers of neuropathic GD mutations (1.4% of patients) had hazard ratios (HRs) for global cognitive impairment of 3.17 (95% confidence interval [CI], 1.60–6.25) and a hastened decline in Mini–Mental State Exam scores compared to noncarriers (*p* = 0.0009). Carriers of complex *GBA* alleles (0.7%) had an HR of 3.22 (95% CI, 1.18–8.73; *p* = 0.022). By contrast, the common, non‐neuropathic N370S mutation (1.5% of patients; HR, 1.96; 95% CI, 0.92–4.18) or nonpathogenic risk variants (6.6% of patients; HR, 1.36; 95% CI, 0.89–2.05) did not reach significance.

**Interpretation:**

Mutations in the *GBA* gene pathogenic for neuropathic GD and complex alleles shift longitudinal cognitive decline in PD into “high gear.” These findings suggest a relationship between specific types of *GBA* mutations and aggressive cognitive decline and have direct implications for improving the design of clinical trials. Ann Neurol 2016;80:674–685

Initial motor features of Parkinson's disease (PD) typically respond to dopaminergic medications. Dopaminergic therapies do not slow the underlying neurodegenerative disease process. Over time, the neuropathology spreads, and, in many patients, dementia emerges as one of the most debilitating and intractable complications of the disease.^1^ The pace of this cognitive disease progression varies considerably between patients. Information on genes that predict prognosis (and modulate disease progression) is needed, both for improving trial design, especially with respect to disease modifying therapies, as well as for developing a personalized medicine.

We hypothesized that mutations in the glucocerebrosidase gene (*GBA*) enriched in severe, neuropathic Gaucher's disease (GD), but not those typical of mild, non‐neuropathic GD, will be associated with an aggressive cognitive decline in PD. Two mutant copies of *GBA* cause GD, the most prevalent lysosomal storage disease. This autosomal‐recessive disorder is linked to over 300 pathogenic mutations in the *GBA* gene,^2^ which encodes β‐glucocerebrosidase. Severity of the GD phenotype varies dramatically. Some GD patients have peripheral manifestations without neurological impairment (non‐neuropathic type 1; eg, hepatosplenomegaly, anemia, and bone disease).^3^ Others show early‐onset, rapidly progressive neurological disease (neuropathic type 2) or a spectrum of chronic neurological manifestations (subacute neuropathic type 3).^3^ Chronic neurologic manifestations of GD include eye movement and motor abnormalities, ataxia, spasticity, seizures, as well as tremor. The current nosology for GD is primarily based on the categorical presence or absence of central nervous system disease and has clinical utility, although considerable variation exists.^3^

Individuals carrying one mutant copy of *GBA* do not develop GD. Heterozygotes, however, are 5‐fold increased among patients with PD,^4^ and mutations in the *GBA* have emerged as the most common protein‐coding risk variants for PD.^5^ *GBA* mutations are also associated with dementia with Lewy bodies.^5^ Initial observations in 15,^6^ 13,^7^ ∼6,^8^ and, recently, 19 carriers^9^ have suggested that—overall—*GBA* mutations may be associated with the rate of progression of PD.^6^, ^7^, ^8^ However, these studies could not deconvolute the specific effects of neuropathic and non‐neuropathic types of *GBA* mutations on progression phenotypes because of the limited numbers of carriers included. For stratification in clinical trials or proactive interventions (eg, designed to prevent dementia in patients with PD) it is important to have the ability to predict the disease course of individual patients. It is thus of practical importance to precisely understand the relation between specific types of *GBA* mutations and the speed of PD progression. Here, we determined that *GBA* mutations linked to neuropathic GD, but not those of non‐neuropathic GD, are associated with a more rapid longitudinal cognitive decline in seven international cohorts representing 2,304 patients with PD longitudinally evaluated for up to 12.8 years (median, 4.1) with 20,868 study visits.

## Subjects and Methods

### Study Participants and Procedures

Seven longitudinal cohorts^6^, ^10^, ^11^, ^12^, ^13^, ^14^, ^15^, ^16^ from North America and Europe representing 2,304 patients with PD (and available DNA) were analyzed (Table 1). The analysis included two population‐based, incident cohort studies (Cambridgeshire Parkinson's Incidence from GP to Neurologist [CamPaIGN],^17^ Parkinsonism: Incidence, Cognition and Non‐motor heterogeneity in Cambridgeshire (PICNICS)^14^, ^18^; five purpose‐built biomarkers and clinical observational studies from academic centers (Harvard Biomarker Study [HBS],^19^, ^20^, ^21^, ^22^ PROfiling PARKinson's disease [PROPARK],^16^ and the French Drug Interaction with Genes in PD [DIGPD]); as well as two well‐phenotyped, failed phase III clinical trials with longitudinal, observational extension studies (Deprenyl and Tocopherol Antioxidative Therapy of Parkinsonism [DATATOP]^15^; Parkinson Research Examination of CEP‐1347 Trial/A Longitudinal Follow‐up of the PRECEPT Study Cohort [PreCEPT/PostCEPT]^13^. Six cohorts enrolled patients with a diagnosis of PD established according to modified UK PD Society Brain Bank diagnostic criteria. In DATATOP, the eligibility criteria required a diagnosis of early, idiopathic PD (Hoen & Yahr [HY] stages 1 or 2) not on antiparkinsonian medications^23^. Detailed eligibility criteria for the cohorts have been previously reported.^10^, ^13^, ^14^, ^16^, ^22^, ^23^, ^24^, ^25^ For all cohorts, diagnostic certainty was increased by confirming the clinical diagnosis of PD during longitudinal follow‐up visits.^26^ In PRECEPT, the diagnosis was supported by neuroimaging. In the DATATOP, the diagnosis was further informed by record reviews and autopsies. Patients identified during follow‐up whose evaluations were not consistent with a diagnosis of PD were excluded from analysis. Written informed consent was obtained from all subjects under the supervision of each local ethics committee. Patients with a known *LRRK2* G2019S mutation were excluded.

**Table 1:** Overview of Study Cohorts

Study (Country)	N (% male)	Age at Enrollment (years, SD)	Years of Education (years, SD)	Study Years (years, range)	Mutation No. of Subjects (%)	Mutations Screened
HBS (USA)					42 (7.6)	Targeted sequencing or N370S, E326K, T369M genotyping
Carriers	42 (59.5)	65.2 (10.2)	15.0 (1.7)	1.7 (0.0–5.2)		
Noncarriers	514 (64.6)	66.1 (9.8)	15.1 (1.9)	1.8 (0.0–8.0)		
DATATOP (USA, Canada)					38 (8.7)	Targeted sequencing
Carriers	39 (51.3)	61.1 (8.1)	13.6 (3.2)	6.6 (0.0–7.6)		
Noncarriers	398 (68.1)	60.0 (9.1)	14.3 (3.4)	6.3 (0.0–7.8)		
DIGPD (France)					32 (7.8)	Sanger sequencing
Carriers	32 (50.0)	60.9 (8.9)	11.3 (3.3)	2.5 (0.0–5.0)		
Noncarriers	377 (59.4)	62.5 (9.8)	12.1 (3.3)	2.2 (0.0–5.0)		
CamPaIGN (UK)					15 (13.2)	Sanger sequencing
Carriers	15 (73.3)	67.1 (9.4)	10.5 (2.6)	6.3 (0.0–11.8)		
Noncarriers	99 (54.5)	69.8 (9.9)	11.7 (3.4)	7.0 (0.0–12.8)		
PICNICS (UK)					8 (6.2)	Sanger sequencing
Carriers	8 (62.5)	63.8 (7.8)	12.5 (2)	3.7 (0.0–4.7)		
Noncarriers	121 (66.1)	69.2 (9.2)	12.1 (2.9)	3.0 (0.0–6.7)		
PROPARK (Netherlands)					53 (16.2)	Targeted sequencing or whole‐exome sequencing
Carriers	53 (69.8)	58.9 (10.1)	12.2 (4.4)	4.5 (0.0–5.4)		
Noncarriers	274 (65.3)	59.7 (10.9)	12.0 (4.1)	4.7 (0.0–6.3)		
PreCEPT (USA, Canada)					32 (9.6)	Targeted sequencing
Carriers	32 (56.3)	58.5 (7)	15.8 (3.4)	6.7 (0.0–8.2)		
Noncarriers	300 (67.7)	60.7 (9.6)	16.1 (3.1)	6.7 (0.0–8.6)		

The study names are Harvard Biomarkers Study (HBS)^10^, ^21^, ^22^; Deprenyl and Tocopherol Antioxidative Therapy of Parkinsonism (DATATOP)^15^, ^23^; Parkinson Research Examination of CEP‐1347 Trial/A Longitudinal Follow‐up of the PRECEPT Study Cohort (PreCEPT/PostCEPT)^13^; Cambridgeshire Parkinson's Incidence from GP to Neurologist (CamPaIGN)^6^, ^11^, ^24^; Parkinsonism: Incidence, Cognition and Non‐motor heterogeneity in Cambridgeshire (PICNICS)^14^; Drug Interaction with Genes in PD (DIGPD)^25^; and PROfiling PARKinson's disease (PROPARK) study.^16^ HBS was examined in two parts: 383 participants for whom targeted genotyping of three *GBA* mutations was performed; 173 individuals for whom full sequencing of the *GBA* locus was performed (targeted sequencing).

Mutations were identified through targeted next‐generation sequencing of the entire *GBA* coding sequence and flanking intronic regions in four data sets (Table 1). For 173 PD samples in HBS, 332 PD samples in PreCEPT/PostCEPT and 437 PD samples in DATATOP, as well as 164 PD samples from PROPARK, *GBA* mutations were systematically identified through full sequencing of the exons and flanking intronic regions of *GBA* in RefSeq (NM_001005741.2). To avoid sequencing its neighboring pseudogene, the entire locus was amplified in a single long‐range polymerase chain reaction (PCR) reaction using the LA PCR Kit (version 2.1; Takara Bio Inc., Otsu, Japan). Template DNA (100ng) was added to a 50‐µl reaction along with primers (final concentration, 0.4 µM) with the following sequences: forward primer (5′‐CGACTTTACAAACCTCCCTG‐3′) and reverse primer (5′‐CCAGATCCTATCTGTGCTGG‐3′), and cycling conditions were 94°C for 1 minute (one cycle), 98°C for 10 seconds followed by 68°C for 15 minutes (30 cycles), and 72°C for 10 minutes (one cycle). This long‐range PCR assay uses primers that target sequences that uniquely flank *GBA* and produces a single 7,755‐base‐pair (bp) PCR product. PCR products were visualized on an 0.8% agarose gel with ethidium bromide to confirm successful amplification, which were then used to construct Illumina ready sequencing libraries using the NexteraXT kit (Illumina Inc., San Diego, CA), following the manufacturer's instructions. Uniquely indexed samples were pooled (up to 384 samples/pool) and run on the Illumina MiSeq instrument to generate 150‐bp paired‐end reads. Sequencing reads were aligned to the human assembly genome (GRCh37/hg19) using BWA^27^ (version 0.6.1). *GBA* mutations were called by the GATK^28^ (version 1.6) toolkit. A genotype quality of at least 50 and at least 10 × coverage was achieved for all samples. As a quality control, reproducibility of mutation detection was assessed by sequencing 57 samples across different batches in replicates, and the concordance rate was 100% across the *GBA* mutation locus for all replicates. For 383 PD sample in HBS, E326K, N370S, and T369M were previously genotyped.^21^ Genotypes for 339 of the 383 samples were confirmed on the Illumina NeuroX chip with a genotyping concordance rate of 100%; for 114 PD samples in CamPaIGN and 129 PD samples in PICNICS, mutations and common genetic variants had been identified through full exonic sequencing of *GBA* after two‐stage PCR, as part of a previous study.^6^ For 409 samples in DIGPD, exons and flanking intronic regions of *GBA* were sequenced. To avoid amplifying and sequencing the neighboring pseudogene, *GBA* was amplified in three large fragments (a 2,972‐bp fragment encompassing exons 1–5; a 2,049‐bp fragment encompassing exons 5–7, and a 1,682‐bp fragment encompassing exons 8–11), using previously described primers and a unique 648C to 548C touch‐down PCR program.^29^ PCR products were sequenced with internal primers, adjacent to coding exons and exon‐intron boundaries, using the Big Dye Terminator Cycle Sequencing Ready Reaction kit (Applied Biosystems, Foster City, CA), as prescribed. Sequencing products were purified using the Big Dye XTerminator Purification kit (Applied Biosystems), then electrophoresed on an ABI 3730 automated sequencer and analyzed with DNA Sequencing Analysis (version 5.1) and Seqscape (version 2.6) software (Applied Biosystems). One hundred sixty‐three samples from PROPARK, which are part of a larger PD exome cohort belonging to the International Parkinson's Disease Genomics Consortium, were sequenced using the EZ Exome Library v2.0 (Roche NimbleGen, Madison, WI) targeting 44.1Mb. Sequencing reads were aligned to the human reference genome (hg19) with BWA.^27^ Single‐nucleotide variants and small insertions/deletions (indels) were called and filtered using the GATK (version 3.x).^28^ For 91 of the 163 samples, the BAM files were available and calculation of *GBA* coverage was performed. On average, 99.5% of the exonic *GBA* regions were covered for at least 15×. For the remaining samples, the overall targeted exome coverage (10×) is 90.8%.

### Study Design and Statistical Analysis

Differences in continuous and categorical baseline characteristics were compared between noncarriers and carriers of genetic variation in *GBA* (all carriers, carriers of the non‐neuropathic N370S mutation, and carriers of neuropathic GD mutations, respectively), with Student *t* tests, and distribution‐free Mann–Whitney *U* or Fisher's exact tests, as appropriate.

The primary aim of this study was to analyze the effect of four types of *GBA* mutations on cognitive progression in PD. To characterize how distinct types of *GBA* mutations are associated with longitudinal disease progression in PD, we compared four operationally defined groups of PD patients with distinct types of *GBA* mutations to patients with PD not carrying a *GBA* mutation. Patients with PD carrying one of four types of *GBA* mutations or variant were considered and compared to patients with PD not carrying a *GBA* mutation (noncarriers), respectively. ***1**, Carriers of a GBA risk variant*. The E326K, T369M, and E388K variants are associated with risk of PD^21^, ^30^ and are linked to GD when occurring in conjunction with other *GBA* mutations, but it is controversial whether they are per se pathogenic for GD.^31^ ***2**, Carriers of the common, non‐neuropathic N370S mutation*. The pathogenic N370S mutation is classically associated with mild, non‐neuropathic phenotypes of GD type 1. ***3**, Carriers of a neuropathic GD mutation*. For the purpose of this study, patients with PD who were heterozygous carriers of a pathogenic *GBA* mutation associated with neuropathic GD subtypes 2 or 3 in one or more published reports (found on PubMed searches in September 2015) were operationally defined as “carriers of a neuropathic GD mutation” (Supplementary Table 1). This included carriers of the L444P mutation, the prototypical mutation associated with neuropathic GD types 2 and 3. ***4**, Carriers of complex GBA alleles*. Patients carrying complex *GBA* alleles (eg, more than one *GBA* mutation/variant) were grouped separately. We also explored longitudinal cognitive decline in patients with PD carrying any pathogenic *GBA* mutation or risk variant taken together (“all *GBA* carriers”) compared to patients with PD not carrying a *GBA* mutation.

Generalized longitudinal mixed fixed‐ and random‐effects analysis of cognitive decline was performed^32^ using serial Mini–Mental State Exam (MMSE) scores longitudinally observed in all cohorts starting from study enrollment. Ninety‐five percent of visits (19,801 of 20,868 study visits) were conducted within 6.5 years from the enrollment visit (median follow‐up period, 4.1 years; maximum follow‐up period, 12.8 years). The MMSE score was the dependent variable and the primary predictors were carrier status, time in the study (years), and their interaction. Fixed covariates were sex, age, and disease duration upon enrollment and years of education. An intercept term and linear rate of change across time per subject were the random terms (permitted to be correlated). A study term was also included as a random effect. To avoid problems with somewhat non‐normal residuals, *p* values were obtained by penalized quasi‐likelihood ratio tests of the full model with the effect in question contrasted with the model without the effect in question. These analyses were implemented in R^33^ (version 3.1.2; R Foundation for Statistical Sciences) using the glmmPQL function in the MASS package (version 7.3‐40). *p* values less than or equal to 0.05 were considered statistically significant.

Cox proportional hazards models were used to estimate the influence of carrier status on the hazard ratio of time to the endpoint reaching global cognitive impairment as indicated by a MMSE ≤25 according to the recommendation the International Parkinson and Movement Disorder Society (MDS) Task Force.^34^ Age at onset was defined as the patient's age at the time of PD diagnosis for six studies. PROPARK defined age at onset as the patient's age at the time of first patient‐reported motor symptoms. A total of 63.7% of patients (1,467 of 2,304) were enrolled into a study cohort within 2 years from their onset age, and 95% of study visits fell within 10 years since onset (19,840 of 20,868 visits). For exploratory analysis of motor progression, we evaluated the HR of time to reaching advanced PD operationally defined as reaching an HY stage 3 (bilateral disease with loss of postural balance). One hundred eighty‐eight patients with global cognitive impairment at enrollment and 244 with HY stage 3 already reached at enrollment were removed from the Cox proportional hazards analyses, respectively. Multivariate Cox regression analysis was performed for each study separately and then across studies with carrier status, age at onset of PD, sex, and years of education as predictors of global cognitive impairment and the same except for education as predictors of HY stage 3. For the meta‐analysis across studies, a “study” term was included as a random effect (a “frailty” model). The proportional hazards assumption of the Cox regression model was tested and not violated by any predictor in any analysis. Cochran's Q‐test was used to test for heterogeneity of effects across studies.

### Hypothetical Power Analysis for a Personalized Clinical Trial Targeting Neuropathic GBA‐PD

To estimate sample‐size requirements for a personalized, 3‐year clinical trial of a hypothetical drug designed to halt cognitive decline (as measured by the MMSE) specifically in patients carrying a neuropathic *GBA* mutation, we ran a power analysis. We used a repeated‐measures analysis of variance design of two groups versus four time points (enrollment, 1 year, 2 years, and 3 years in study). One hypothetical group was assigned to placebo and therefore stipulated to have MMSE means across time predicted by our mixed‐effects model, and the second group was assigned to treatment with an experimental drug, which has the hypothetical ability to halt decline in MMSE scores (all scores set equal to the predicted MMSE scores at enrollment). We ran analyses assuming a two‐tailed α of 0.05 to detect the difference in trajectories across time for the two groups (group × time interaction), and assuming a within group/time‐point standard deviation (SD) of 2 and a 1‐year test‐retest correlation of 0.7 as approximate to those values found empirically, with a first‐order autoregressive decay across longer periods. The conservative Greenhouse‐Geisser correction for degrees of freedom for correlated error was also used. For comparison, analogous computations were performed for a hypothetical clinical trial scenario with “all comer” PD patients (not carrying a *GBA* mutation).

We found required sample sizes to be 36 per placebo and 36 per drug group in order to achieve 80% power. By contrast, if instead all‐comer patients with PD (not carrying a *GBA* mutation) were enrolled to test the same experimental drug (over the same time period, assuming same α, same SD, and same test‐retest correlations), 893 patients would be required per placebo and 893 per drug group to achieve 80% power (Fig 3). Thus, a trial targeted to neuropathic *GBA*‐PD could shrink sample‐size requirements by as much as 25‐fold compared to an equally powered trial of all‐comer PD patients without a *GBA* mutation.

## Results

### Clinical Cohort Characteristics at Enrollment

Mean ages at enrollment across the seven cohorts ranged from 59.6 to 69.4, and mean MMSE scores from 27.1 to 29.3. Mean HY stages at enrollment ranged from 1.0 to 2.6.

### GBA Mutations

**Figure 1:**
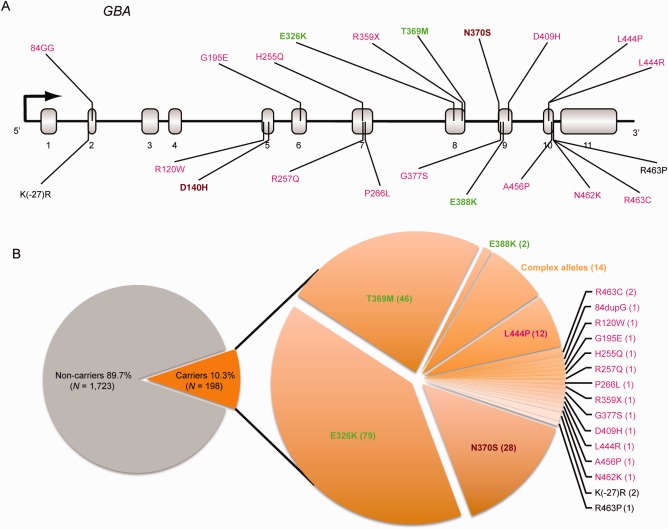
Distribution of mutations in the Gaucher's disease gene *GBA* among study patients with Parkinson's disease. Overall, 10.3% of the 1,921 patients with Parkinson's analyzed using sequencing were carriers of a *GBA* mutation. The location of *GBA* mutations identified in this study is shown in (A) (RefSeq NM_001005741.2). Mutations reported in neuropathic GD type 2 or 3 are shown in magenta font. Mutations associated with mild, non‐neuropathic GD type 1 (eg N370S) are shown in brown font. Risk variants are shown in green, and those variants, whose clinical phenotype is not established, in black font. The distribution of mutations is shown in the pie chart to the right of (B) with the number of carriers observed in parenthesis. GD = Gaucher's disease.

Overall, 10.3% of the 1,921 patients with PD analyzed by sequencing were carriers of a *GBA* mutation (Fig 1). A total of 1.4% (26) of patients were heterozygous carriers of a neuropathic GD mutation (Fig 1). These included the L444P mutation (found in 12 heterozygous carriers), as well as the 84GG, G195E, H255Q, R257Q, P266L, R359X, G377S, D409H, L444R, A456P, N462K, R120W, and R463C mutations (Supplementary Table 1). A total of 1.5% (28) were carriers of the non‐neuropathic N370S mutation. A total of 6.6% (127) were heterozygous carriers of a risk variant (79 were carriers of the E326K, 46 with T369M, and 2 with E388K risk variants). A total of 0.7 % (14) were carrier of a complex *GBA* allele. Three additional rare mutations (D140H, K(‐27)R, and R463P) were detected. The R463P and K(‐27)R mutations have not been conclusively associated with a GD subtype and were thus excluded from the mutation‐type specific analyses. D140H mutations identified were part of complex alleles (Fig 1; Supplementary Table 1).

### Patients With PD Carrying GBA Mutations Enriched in Neuropathic GD Had an Aggressive, Accelerated Longitudinal Cognitive Decline

At enrollment, disease duration, age at onset, and levodopa equivalent drug dose were not materially different in carriers of a neuropathic *GBA* mutation compared to noncarriers included in the proportional hazards model analysis (Table 2). Importantly, for this analysis, at enrollment, MMSE scores were identical for carriers of a neuropathic GD mutation compared to noncarriers; their motor scores (HY stage and MDS Unified Parkinson's Disease Rating Scale Part III) at enrollment were slightly elevated (Table 2).

**Table 2:** Clinical Characteristics of Participants With PD at Enrollment

N = 2,116	Noncarriers	All *GBA* Carriers	*p* *	Carriers of Non‐neuropathic N370S	*p* **	Carriers of Neuropathic GD Mutations	*p* ***
Total No. (N)	1,918	198		38		24	
No. of men (N, %)	1,242 (64.6)	115 (58.1)	0.07	24 (63.2)	0.86	9 (37.5)	0.01
No. of Europeans (N, %)^a^	1,602 (83.5)	150 (75.8)	0.01	32 (84.2)	1.00	19 (79.2)	0.58
Age of onset (years, SD)	60.1 (10.6)	58.4 (9.7)	0.02	59.3 (8.9)	0.56	59.7 (10.9)	0.83
Disease duration at enrollment (years, SD)	2.6 (3.4)	2.6 (3.7)	0.96	2.0 (2.3)	0.13	2.0 (3.3)	0.36
Years of education (years, SD)	14.1 (3.4)	13.6 (3.6)	0.07	13.7 (3.2)	0.49	13.8 (3.9)	0.77
**MMSE (mean, SD)**	**28.8 (1.2)**	**28.8 (1.2)**	**0.97**	**28.8 (1.1)**	**0.89**	**29.2 (1.0)**	**0.17**
MDS‐UPDRS III (mean, SD)	27.7 (13.1)	29.4 (12.9)	0.06	23.9 (12.3)	0.06	32.2 (10.5)	0.04
Hoehn & Yahr stage (mean, SD)	1.8 (0.7)	1.9 (0.7)	0.03	1.7 (0.7)	0.49	2.1 (0.6)	0.02
Levodopa equivalent drug dose^b^ (mean, SD)	330.5 (393.3)	375.5 (430.9)	0.27	403.2 (377.1)	0.09	481.2 (612.8)	0.28

Group comparisons were performed using Student *t* test for age at enrollment, age at onset, and disease duration at enrollment; Mann‐Whitney‐Wilcoxon test for MDS‐UPDRS, Hoehn & Yahr, MMSE, and levodopa equivalent drug dose; and the Fisher's exact test for sex and ancestry. Note that at enrollment, mean MMSE scores for carriers and non‐carriers were virtually identical (bold).

* All carriers compared to noncarriers; **carriers of the non‐neuropathic N370S mutation compared to noncarriers; ***neuropathic GD mutation carriers compared to noncarriers.

a Ancestry information was not available for PROPARK.

Levodopa equivalent drug dose was not available for the PreCEPT/PostCEPT cohort.

**Figure 2:**
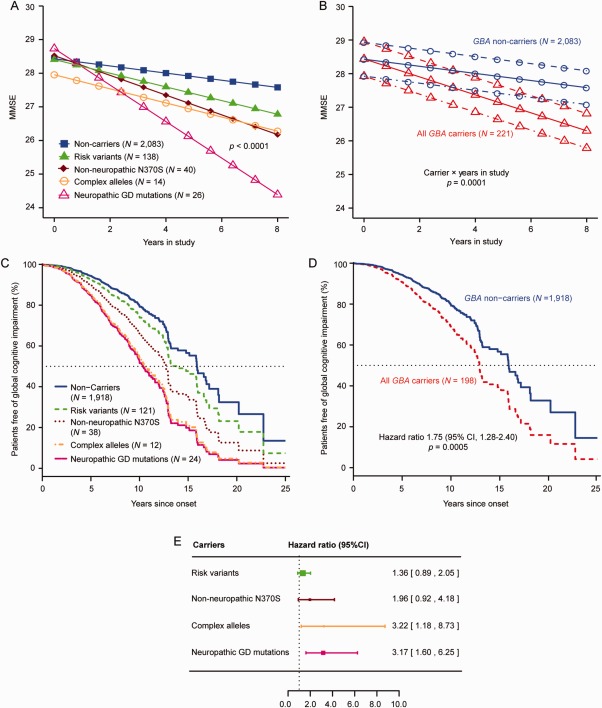
Specifically neuropathic GD mutations accelerate cognitive decline in patients with PD. (A,C,E) Neuropathic GD mutations carriers were linked to a more‐rapid cognitive decline in PD (in heterozygotes). (A) Neuropathic GD mutations predicted decline in Mini–Mental State Exam scores over time in the generalized longitudinal mixed model meta‐analysis in heterozygous patients. Illustrative mean scores on the MMSE across time predicted from the estimated fixed‐effect parameters in the mixed random‐ and fixed‐effects model analysis are shown for Parkinson's patients carrying specific types of *GBA* mutations and those without a *GBA* mutation. Carriers of a neuropathic GD mutation showed accelerated longitudinal cognitive decline compared to noncarriers with *p* < 0.0001, adjusting for the covariates of age at enrollment, sex, duration of PD upon enrollment, and years of education. Illustrative mean scores on the MMSE across time for PD patients with a neuropathic GD mutation are shown as magenta triangles; values for PD patients without a *GBA* mutation are represented as blue squares. Illustrative means scores on the MMSE across time for carriers of a risk variant (heterozygous carriers of E326K, T369M, and E388K; green triangles), the non‐neuropathic N370S mutation (brown rhombi), or complex *GBA* alleles (orange circles) are also shown. (B) Illustrative mean MMSE scores across time predicted from the estimated fixed‐effect parameters in the mixed random‐ and fixed‐effects model analysis are shown for Parkinson's patients without a *GBA* mutation (noncarriers) and those carrying any of the *GBA* mutations (all carriers). Carriers had overall a more‐rapid decline in cognitive function (as measured by serial MMSE) compared to noncarriers with *p* = 0.0001, after adjusting covariates (solid lines indicate mean value of disease duration at enrollment; dotted‐dashed lines indicate 1 SD longer disease duration at enrollment; and dashed lines indicate 1 SD shorter disease duration at enrollment). (C) Covariate adjusted survival curves for Parkinson's patients without a *GBA* mutation (noncarriers; blue line) and those carrying specific types of *GBA* mutations: risk variants (green, interrupted line), the common, non‐neuropathic N370S mutation (brown, dotted line), neuropathic GD mutations (magenta line), or complex *GBA* alleles (orange, dotted‐dashed line). (D) All carriers of a *GBA* mutation, taken together, had an overall hazard ratio for global cognitive impairment of 1.75 (95% CI, 1.28–2.40) compared to noncarriers with *p* = 0.0005, adjusting for age of onset, sex, years of education, and study. The covariate adjusted survival curves are shown (carriers, red interrupted line; noncarriers, blue line). The means of covariate‐adjusted predicted values are visualized. (E) The forest plot shows hazard ratios for global cognitive impairment in carriers of one of these specific types of *GBA* mutations. The hazard ratio for global cognitive impairment in carriers of neuropathic GD mutation was 3.17 (95% CI, 1.60–6.25; magenta). The squares represent point estimates, with the height of the square inversely proportional to the standard error of the estimates. The horizontal lines indicate 95% confidence intervals of the estimates. In (A), the group of patients with neuropathic GD mutations includes 26 heterozygous carriers with the following mutations: 12 with L444P, 2 with R463C, and 1 each of R257Q, 84dupG, R120W, D409H, R359X, P266L, N462K, A456P, L444R, G377S, H255Q, and G195E. The 14 carriers of complex alleles shown in (A) included 8 patients with E326K and D140H, 1 with E326K and T369M, 1 with E326K and R463C, and 1 with E326K and R257Q; and homozygous carriers of E326K/E326K, T369M/T369M, and E326/E326K/L444P/L444P genotypes, respectively. In (C), the group of patients with neuropathic GD mutations includes 24 heterozygous carriers with neuropathic GD mutations: 12 with L444P, 2 with R463C, and 1 each of R257Q, 84dupG, R120W, R359X, P266L, A456P, L444R, G377S, H255Q, and G195E. The 12 carriers of complex alleles shown in (C) included 6 patients with E326K plus D140H mutations, 1 with E326K plus T369M, 1 with E326K plus R463C, and 1 with E326K plus R257Q; and homozygotes carriers with E326K/E326K, T369M/T369M, and E326/E326K/L444P/L444P, respectively. It should be note that in the Cox proportional hazards analyses, the number of mutation carriers differs from that in the mixed fixed‐ and random‐effects analysis, attributed to the removal of subjects, who had already reached the endpoint at enrollment (left censored). The number of mutation carriers available for this analysis also differs from the number of carriers shown in Figure 1 because partially genotyped samples were here included, whereas data only for fully sequenced samples are shown in Figure 1. CI = confidence interval; GD = Gaucher's disease; SD = standard deviation.

We performed a generalized mixed random‐ and fixed‐effects longitudinal meta‐analysis for MMSE scores, adjusting for the covariates of age at enrollment, sex, duration of PD at enrollment, and years of education. Neuropathic GD mutations predicted a significant decline in MMSE scores over time with *p* < 0.0001 (Fig 2A).

**Figure 3:**
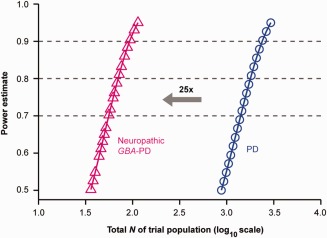
Improved power in genetically targeted clinical trials. A trial targeted to neuropathic *GBA*‐PD could shrink sample size requirements by as much as 25‐fold compared to an equally powered trial of “all comers” PD patients (without a *GBA* mutation) in this hypothetical power estimate. Required sample sizes were 36 for the placebo and 36 for the experimental treatment group in order to achieve 80% power. A traditional clinical trial of “all comers” PD patients (not carrying a *GBA* mutation) would require 893 patients *per* group to achieve the same power (over the same 3‐year time period, assuming same α, standard deviation, and test‐retest correlations). α = 0.05 for detecting the difference in trajectories for MMSE across time for the placebo versus the treatment group (group × time interaction), MMSE scores predicted by our study were used. See Methods for details. MMSE = Mini–Mental State Exam; PD = Parkinson's disease.

Carriers of a neuropathic *GBA* mutation had a Cox proportional HR for global cognitive impairment of 3.17 (95% confidence interval [CI], 1.60–6.25) compared to noncarriers (*p* = 0.0009), adjusting for age of onset, sex, years of education, and study (Fig 2C,E). At 10 years from diagnosis, 79.5% (95% CI, 76.4–82.8) of noncarriers were free of global cognitive impairment compared to 52.2% (95% CI, 33.9–80.5) of neuropathic GD mutation carriers (a 27.3% difference).

### Patients with PD Carrying Complex GBA Alleles Also Had a More Rapid Longitudinal Cognitive Decline

Carriers of complex *GBA* alleles had a Cox proportional HR for global cognitive impairment of 3.22 (95% CI, 1.18–8.73) compared to noncarriers (*p* = 0.022), adjusting for age of onset, sex, years of education, and study (Fig 2C,E). In the mixed random‐ and fixed‐effects model analysis, the decline in MMSE scores was somewhat less pronounced, likely due to lower MMSE scores at enrollment recorded for this group of patients (Fig 2A).

### Cognitive Decline in Patients With PD Carrying the Non‐Neuropathic N370S Mutation Was Not Materially Faster Than in Noncarriers

Carriers of the non‐neuropathic N370S mutation alleles had a Cox proportional HR for global cognitive impairment of 1.96 (95% CI, 0.92–4.18) compared to noncarriers, adjusting for age of onset, sex, years of education, and study (Fig 2C,E).

Patients with PD carrying *GBA* risk variants had a Cox proportional HR for global cognitive impairment of 1.36 (95% CI, 0.89–2.05) compared to noncarriers, adjusting for age of onset, sex, years of education, and study (Fig 2E).

*GBA* mutations predicted a decline in MMSE scores over time (Fig 2B) in all carriers compared to noncarriers with *p* = 0.0001 in the generalized mixed random‐ and fixed‐effects model analysis, adjusting for covariates and study. Multiple lines of evidence, including sensitivity analyses,^35^ indicated that dropout bias did not unduly influence this analysis. All carriers of a *GBA* mutation, taken together, had a Cox proportional HR for global cognitive impairment of 1.75 (95% CI, 1.28–2.40) compared to noncarriers (*p* = 0.0005), adjusting for age of onset, sex, years of education, and study (Fig 2D). The HRs for global cognitive impairment in mutation carriers versus noncarriers from the Mantel‐Haenszel procedure were examined for each of the seven independent cohorts. Proportional HRs across studies were homogeneous with I^2^ = 40.5%, *p* = 0.21 by Cochran's Q‐test for heterogeneity, suggesting it was not imperative, though permissible, to analyze “studies” as a random term in order to allow a more‐universal inference.

We then explored the effect of *GBA* mutations on motor disease progression. HY stage 3 marks the transition from mild to moderate disease with impaired balance. It is meaningful for patients because of fall risk and impact on quality of life.^36^ In this population, none of the four types of *GBA* mutations investigated was statistically significantly associated with progression to HY stage 3 compared to noncarriers, respectively, in the Cox proportional hazards model adjusted for sex and age at onset. For all carriers of a *GBA* mutation, taken together, the HR for progressing to HY stage 3 was increased by a factor of 1.26 compared to noncarriers. However, this missed the threshold for statistical significance (95% CI, 0.98–1.61; *p* = 0.068).

## Discussion

Seven deeply phenotyped, longitudinal cohorts from North America and Europe representing 2,304 patients with PD were followed longitudinally for up to 12.8 years (median, 4.1) with a total of 20,868 in‐person study visits. This makes the current analysis one of the largest longitudinal observational studies reported for PD. Much progress has been made in delineating genome variation associated with susceptibility for developing PD,^25^, ^37^ but little is known about the genetic architecture controlling disease progression.^11^, ^24^, ^38^ In a surprising analogy to GD, *GBA* mutations enriched in neuropathic GD in homozygotes (leading to death at age 2 or severe neurological complications) were associated with aggressive cognitive decline in PD. Patients carrying these mutations had an HR of 3.17 (95% CI, 1.60–6.25) for developing global cognitive impairment compared to patients without a mutation — a 217% risk increase. By contrast, patients carrying the common N370S mutation linked to non‐neuropathic GD showed a trend toward intermediate rates of longitudinal cognitive decline. This did not reach statistical significance, likely due to insufficient power. This report is the first to evaluate the differential effects of distinct types of neuropathic, complex alleles, non‐neuropathic, and risk variation in *GBA* on the longitudinal trajectory of cognitive decline in PD. It suggests the clinical‐genetic concept of aggressive, neuropathic *GBA*‐PD. The cross‐sectional observation that PD is more frequent among carriers of a neuropathic GD mutations, compared to carriers of non‐neuropathic GD mutations in an Ashkenazi‐Jewish population,^39^, ^40^ is fully consistent with the new longitudinal trajectories here discovered.

This concept has implications for trial design. Enrolling patients with neuropathic *GBA*‐PD into genetics‐guided, proof‐of‐concept trials for disease‐modifying precision therapeutics may increase power, shorten trial duration, shrink sample sizes, and, possibly, cut costs. Our hypothetical power analysis estimates that a trial targeted to neuropathic *GBA*‐PD could shrink the sample size required by as much as 25‐fold compared to an equally powered trial of PD patients without a *GBA* mutation (Fig 3). Furthermore, the MMSE used in the current analysis has limited sensitivity.^41^ More‐sensitive instruments, such as the Montreal Cognitive Assessment scale,^41^ might detect cognitive changes in carriers of a neuropathic GD mutation earlier and further improved statistical power and shorten trial duration. Importantly, prospective interventions (eg, designed to prevent dementia in patients with PD) or therapies with significant side effects require accurate risk‐benefit analyses based on the prognosis for individual patients. Can enough patients with PD and a neuropathic GD mutation be recruited for clinical trials? Neuropathic GD mutations are as common in “sporadic” PD than *LRRK2* G2019S mutations.^42^ Considering that 1.4% of patients with PD from academic centers, clinical trials, and population‐based cohorts in the United States, Canada, and Europe with *GBA* sequencing data carried a neuropathic GD mutation and another 0.7% carried a complex *GBA* allele, this appears possible, albeit challenging. Beyond *GBA*, variants in other loci, such as *MAPT* ^11^and *SNCA*,^43^ are emerging that might contribute to modulating cognitive decline. Moreover, demographic, clinical, and environmental factors are likely to influence the rate of cognitive decline in a particular patient (eg, Zhu et al^44^).

This study has considerable strengths. Genetic analyses of the *GBA* locus were performed at the end of the clinical longitudinal follow‐up period. Physicians therefore recruited and longitudinally assessed the participants without knowledge of their *GBA* genotype. This design should be less vulnerable to recruitment and ascertainment bias than previous case‐control studies given that patients were assigned to one of two groups simply based on the presence or absence of mutated *GBA* alleles in a form of “double‐blinded Mendelian randomization.” The meta‐analysis included two community‐based cohorts^6^, ^14^ and six from academic centers with cohort‐specific eligibility criteria, differences in enrollment ages, and recruitment from distinct source populations.

Confirmation across multiple heterogeneous populations likely adds to the validity of the results. Although time‐static “cross‐sectional” studies certainly have their value, longitudinal studies are generally considered more informative and powerful and provide information about individual change.^32^ Most important, of course, is the fact that longitudinal designs examine the critical time dimension, which could be entirely hidden in a cross‐sectional study.

A constraint of this meta‐analysis is that in DATATOP and PreCEPT, DNA was collected several years after enrollment for a subset of participants.^13^ Thus, they may under‐represent patients with more rapidly progressive disease, but it is unlikely that this would yield a spurious association between *GBA* mutations and rapid cognitive decline.

The mechanism through which *GBA* mutations modulate the PD process is controversial.^45^ Autosomal‐recessive mutations in *GBA* cause GD through a decrease or loss in GCase function and replacing GCase enzyme in patients with GD corrects hepatosplenomegaly and hematological abnormalities. L444P, the prototypical mutation of neuropathic GD, leads to reduced β‐glucocerebrosidase (GCase) enzymatic activity.^37^, ^46^ N370S appears to confer a milder reduction in GCase activity.^46^ In PD, however, both *GBA* loss‐of‐function and toxic gain‐of‐function hypotheses have been proposed.^5^, ^47^ In cultured cells, mutant *GBA* promoted α‐synuclein accumulation in a dose‐dependent manner without observed loss of GCase function.^47^ However, in PD patients heterozygous for a *GBA* mutation, GCase activity is reduced in induced pluripotent stem‐cell–derived neurons,^48^ brain,^45^ cerebrospinal fluid,^48^ and blood.^37^ Reduced GCase activity causes accumulation of glucosylceramide,^48^ lysosomal dysfunction,^49^ and accumulation of α‐synuclein protein,^47^, ^48^, ^49^, ^50^ a neuropathological hallmark of PD. Furthermore, α‐synuclein, in turn, may further reduce the availability of functional glucocerebrosidase,^45^, ^49^ and GCase activity is low even in PD patients without a *GBA* mutation, although to a lesser extent than those with a mutation.^45^ Thus, it is possible that therapeutics effective in *GBA*‐PD could also be of benefit for PD patients without a *GBA* mutation.

In GD, enzyme replacement therapy is effective in reversing peripheral disease manifestations, but does not penetrate the blood–brain barrier. In an encouraging development for both neuropathic GD and PD, small molecules designed to correct the GCase pathway in the brain are in development.

## Author Contributions

G.L., B.B., J.J.L., B.R., J.J.v.H., J.‐C.C., R.A.B., J.M., C.H.W.‐G., and C.R.S. were responsible for conception and design of the study. All authors were responsible for acquisition and analysis of data. G.L., B.B., J.J.L., R.A.B., C.H.W.‐G., and C.R.S. were responsible for drafting a significant portion of the manuscript or figures.

## Potential Conflicts of Interest

C.R.S. has a scientific collaboration with Genzyme.

## Supporting information

Additional supporting information can be found in the online version of this article

Supporting Information
