# Supplementary material for: Specifically neuropathic Gaucher's mutations accelerate cognitive decline in Parkinson's
Source: Ann Neurol. 2016 Nov 18;80(5):674–85. doi: 10.1002/ana.24781 (PMC5244667; doi:10.1002/ana.24781)
Supplement: Supplementary file 1 — Supporting Information [file ANA-80-674-s001.docx]

**Neuropathic Gaucher’s mutations accelerate cognitive decline in Parkinson’s**

**SUPPLEMENTARY TABLES**

**CONTENTS**

Contributing members of the International Genetics of Parkinson Disease Progression (IGPP) Consortium and HBS, DIGPD, PreCEPT/PostCEPT, CamPaIGN, PICNICS and PROPARK investigators. 2

Supplemental Table 1: Rare *GBA* mutations identified and their relation to non-neuropathic GD type 1 or neuropathic GD type 2/3. 5

Supplemental Table 2: Carriers of complex *GBA* alleles. 6

| **Contributing members of the International Genetics of Parkinson Disease Progression (IGPP) Consortium** | | | |
| --- | --- | --- | --- |
| **Name** | **Institute** | **City, State** | **Country** |
| Zhixiang Liao | Harvard Medical School and Brigham & Women's Hospital | Boston, MA 02115 | USA |
| Kara Page | Harvard Medical School and Brigham & Women's Hospital | Boston, MA 02115 | USA |
| Daly Franco | Harvard Medical School and Brigham & Women's Hospital | Boston, MA 02115 | USA |
| Karen Duong | Harvard Medical School and Brigham & Women's Hospital | Boston, MA 02115 | USA |
| Tom Yi | Harvard Medical School and Brigham & Women's Hospital | Boston, MA 02115 | USA |
| Ana Trisini-Lipsanopoulos | Harvard Medical School and Brigham & Women's Hospital | Boston, MA 02115 | USA |
| Xianjun Dong | Harvard Medical School and Brigham & Women's Hospital | Boston, MA 02115 | USA |
| Lewis R. Sudarsky | Brigham & Women's Hospital | Boston, MA 02115, | USA |
| Samantha J. Hutten | Michael J. Fox Foundation | New York, NY 10163-4777 | USA |
| Sami S. Amr | Translational Genomics Core of Partners HealthCare Personalized Medicine | Cambridge, MA 02139 | USA |
| Ira Shoulson | Department of Neurology, Georgetown University | Washington, DC 20007 | USA |
| Caroline M. Tanner | San Francisco Veterans Affairs Medical Center & Department of Neurology, UCSF School of Medicine | San Francisco CA 94121 | USA |
| Anthony E. Lang | Toronto Western Hospital and the University of Toronto, | Toronto ON, M5T 2S8 | Canada |
| Mike A. Nalls | Laboratory of Neurogenetics, National Institute on Aging | Bethesda, MD 20892 | USA |

| **Harvard Biomarkers Study (HBS)** | |  |  |
| --- | --- | --- | --- |
| Clemens Scherzer | Brigham & Women's Hospital | Boston, MA 02115 | USA |
| Bradley T. Hyman | Brigham & Women's Hospital | Boston, MA 02115 | USA |
| Adrian J. Ivinson | Brigham & Women's Hospital | Boston, MA 02115 | USA |
| Ana Trisini-Lipsanopoulos | Brigham & Women's Hospital | Boston, MA 02115 | USA |
| Daly Franco | Brigham & Women's Hospital | Boston, MA 02115 | USA |
| Kyle Burke | Brigham & Women's Hospital | Boston, MA 02115 | USA |
| Lewis R. Sudarsky | Brigham & Women's Hospital | Boston, MA 02115, | USA |
| Michael T. Hayes | Brigham & Women's Hospital | Boston, MA 02115 | USA |
| Chizoba C. Umeh | Brigham & Women's Hospital | Boston, MA 02115 | USA |
| John H. Growdon | Massachusetts General Hospital | Boston, MA 02114 | USA |
| Michael A. Schwarzschild | Massachusetts General Hospital | Boston, MA 02114 | USA |
| Albert Y. Hung | Massachusetts General Hospital | Boston, MA 02114 | USA |
| Alice W. Flaherty | Massachusetts General Hospital | Boston, MA 02114 | USA |
| Anne-Marie Wills | Massachusetts General Hospital | Boston, MA 02114 | USA |
| Nicte I. Mejia | Massachusetts General Hospital | Boston, MA 02114 | USA |
| Stephen N. Gomperts | Massachusetts General Hospital | Boston, MA 02114 | USA |
| Vikram Khurana | Massachusetts General Hospital | Boston, MA 02114 | USA |
| Dennis J. Selkoe | Brigham & Women's Hospital | Boston, MA 02115 | USA |
| Thomas Yi | Brigham & Women's Hospital | Boston, MA 02115 | USA |
| Kara Page | Brigham & Women's Hospital | Boston, MA 02115 | USA |
| Zhixiang Liao | Brigham & Women's Hospital | Boston, MA 02115 | USA |

| **Drug Interaction with Genes in Parkinson’s Disease Study (DIGPD)** | | |  |
| --- | --- | --- | --- |
| Jean-Christophe Corvol | Hôpital Pitié-Salpêtrière | F-75013 Paris | France |
| Alexis Brice | Hôpital Pitié-Salpêtrière | F-75013 Paris | France |
| Alexis Elbaz | Hôpital Pitié-Salpêtrière | F-75013 Paris | France |
| Alain Mallet | Hôpital Pitié-Salpêtrière | F-75013 Paris | France |
| Marie Vidailhet | Hôpital Pitié-Salpêtrière | F-75013 Paris | France |
| Anne-Marie Bonnet | Hôpital Pitié-Salpêtrière | F-75013 Paris | France |
| Cecilia Bonnet | Hôpital Pitié-Salpêtrière | F-75013 Paris | France |
| David Grabli | Hôpital Pitié-Salpêtrière | F-75013 Paris | France |
| Andreas Hartmann | Hôpital Pitié-Salpêtrière | F-75013 Paris | France |
| Stephan Klebe | Hôpital Pitié-Salpêtrière | F-75013 Paris | France |
| Lucette Lacomblez | Hôpital Pitié-Salpêtrière | F-75013 Paris | France |
| Graziella Mangone | Hôpital Pitié-Salpêtrière | F-75013 Paris | France |
| Marie Vidailhet | Hôpital Pitié-Salpêtrière | F-75013 Paris | France |
| Frédéric Bourdain | Hôpital Foch (Suresnes): | F-75013 Paris | France |
| Jean-Philippe Brandel | Fondation Rotschild (Paris): | F-75019 Paris | France |
| Pascal Derkinderen | Centre Hospitalo-Universitaire de Nantes | F-44000 Nantes | France |
| Franck Durif | Centre Hospitalo-Universitaire de Clermont-Ferrand | F-63000 Clermont-Ferrand | France |
| Valérie Mesnage | Hôpital Saint-Antoine (Paris) | F-75012 Paris | France |
| Fernando Pico | Centre hospitalier de Versailles | F-78150 Le Chesnay | France |
| Olivier Rascol | Centre Hospitalo-Universitaire de Toulouse | F-31059 Toulouse | France |
| Sylvie Forlani | Hôpital Pitié-Salpêtrière | F-75013 Paris | France |
| Suzanne Lesage | Hôpital Pitié-Salpêtrière | F-75013 Paris | France |
| Graziella Mangone | Hôpital Pitié-Salpêtrière | F-75013 Paris | France |
| Khadija Tahiri | Hôpital Pitié-Salpêtrière | F-75013 Paris | France |

| **Parkinson Research Examination of CEP-1347 Trial/A Longitudinal Follow-up of the PRECEPT Study (PreCEPT/PostCEPT)** | | | |
| --- | --- | --- | --- |
| Bernard Ravina | Voyager Therapeutics | Cambridge, MA 02142 | USA |
| Clemens Scherzer | Harvard Medical School and Brigham & Women's Hospital | Boston, MA 02115 | USA |
| Michael G. Schlossmacher | University of Ottawa | Ottawa, ON K1N 6N5 | Canada |
| Andrew Siderowf | Avid Radiopharmaceuticals | Philadelphia, PA 19104 | USA |
| David Oakes | University of Rochester | Rochester, NY 14642 | USA |
| Kenneth Marek | Institute for Neurodegenerative Disorders | New Haven, CT 06510 | USA |
| Ira Shoulson | Department of Neurology, Georgetown University | Washington, DC 20007 | USA |

| **Cambridgeshire Parkinson’s Incidence from GP to Neurologist (CamPaIGN)** | | | |
| --- | --- | --- | --- |
| Roger Barker | University of Cambridge | Cambridge, CB2 0PY | UK |
| Tom Foltynie | University of Cambridge | Cambridge, CB2 0PY | UK |
| Caroline H. Williams-Gray | University of Cambridge | Cambridge, CB2 0PY | UK |
| Sarah Mason | University of Cambridge | Cambridge, CB2 0PY | UK |
| Sophie Winder-Rhodes | University of Cambridge | Cambridge, CB2 0PY | UK |

| **Parkinsonism: Incidence, Cognition and Non-motor heterogeneity in Cambridgeshire (PICNICS)** | | | |
| --- | --- | --- | --- |
| Roger Barker | University of Cambridge | Cambridge, CB2 0PY | UK |
| Caroline H. Williams-Gray | University of Cambridge | Cambridge, CB2 0PY | UK |
| David Breen | University of Cambridge | Cambridge, CB2 0PY | UK |
| Gemma Cummins | University of Cambridge | Cambridge, CB2 0PY | UK |
| Jonathan Evans | University of Cambridge | Cambridge, CB2 0PY | UK |
| Sophie Winder-Rhodes | University of Cambridge | Cambridge, CB2 0PY | UK |

| **PROfiling PARKinson’s disease study (PROPARK)** | | | |
| --- | --- | --- | --- |
| Jacobus J. van Hilten | Leiden University Medical Center | Albinusdreef 2, 2333 ZA, Leiden | The Netherlands |
| Johan Marinus | Leiden University Medical Center | Albinusdreef 2, 2333 ZA, Leiden | The Netherlands |

# Supplemental Table 1: Rare *GBA* mutations identified and their relation to non-neuropathic GD type 1 or neuropathic GD type 2/3.

| **Number** | **cDNA** | **Allele Name** | **Gaucher’s disease type** | **PubMed ID** |
| --- | --- | --- | --- | --- |
| 1 | c.38A>G | K(-27)R | unknown | 17059888 |
| **2** | **c.84dupG** | **84GG** | **1,2,3** | **1961718** |
| **3** | **c.475C>T** | **R120W** | **1,2** | **8774051** |
| 4 | c.535G>C | D140H | 1 | 2880291 |
| **5** | **c.701G>A** | **G195E** | **1,2** | **9153297** |
| **6** | **c.882T>G** | **H255Q** | **2** | **10649495** |
| **7** | **c.887G>A** | **R257Q** | **1,2,3** | **8790604** |
| **8** | **c.914delC** | **P266L** | **2** | **11783951** |
| **9** | **c.1192C>T** | **R359X** | **1,2** | **8112750** |
| **10** | **c.1246G>A** | **G377S** | **1,3** | **8081401** |
| **11** | **c.1342G>C** | **D409H** | **1,3** | **2269438** |
| **12** | **c.1448T>C** | **L444P** | **1,2,3** | **2880291** |
| **13** | **c.1448T>G** | **L444R** | **2** | **7981693** |
| **14** | **c.1483G>C** | **A456P** | **1,2,3** | **11241841** |
| **15** | **c.1503C>G** | **N462K** | **2** | **9279145** |
| **16** | **c.1504C>T** | **R463C** | **1,3** | **1972019** |
| 17 | c.1505G>C | R463P | unknown | 16185900 |

*GBA* mutations associated with neuropathic Gaucher’s disease type 2 or 3 in at least one previous report are shown in bold font. Mutations exclusively found in non-neuropathic Gaucher’s disease type 1 are shown in regular font.

# Supplemental Table 2: Carriers of complex *GBA* alleles.

| **Number of Patients** | **Complex alleles** | **Allelic location of mutations** |
| --- | --- | --- |
| 7 | E326K/D140H | Unknown |
| 1 | E326K/T369M | Bi-allelic |
| 1 | E326K/R463C | Unknown |
| 1 | E326K/R257Q | Unknown |
| 1 | E326K/E326K | Homozygous, bi-allelic |
| 1 | E326K/E326K, D140H | Homozygous, bi-allelic |
| 1 | T369M/T369M | Homozygous, bi-allelic |
| 1 | E326K/E326K,L444P/L444P | Homozygous, bi-allelic |
